# Midlateral medullary infarction presenting with isolated thermoanaesthesia: a case report

**DOI:** 10.1186/s12883-022-02796-x

**Published:** 2022-07-19

**Authors:** Keisuke Hanada, Kayoko Yokoi, Natsuko Kashida, Ryota Shimomura, Daiki Murata, Kazumi Hirayama

**Affiliations:** 1 Department of Rehabilitation, Suishokai Murata Hospital, Osaka City, Japan; 2 Department of Rehabilitation, Kinsyukai Hanwa Memorial Hospital, 3-5-8, Minami-sumiyoshi, Sumiyoshi ward, Osaka, 558-0011 Japan; 3 Department of Occupational Therapy, Graduate School of Rehabilitation Science, Osaka Metropolitan University, Habikino City, Osaka Japan; 4 Department of Occupational Therapy, Graduate School of Health Sciences, Yamagata Prefectural University of Health Sciences, Yamagata City, Japan; 5 Department of Neurosurgery, Suishokai Murata Hospital, Osaka City, Japan

**Keywords:** Lateral medullary infarction, Lateral spinothalamic tract, Thermal sense, Pain

## Abstract

**Background:**

A small lateral medullary lesion could produce isolated impairment of temperature sensation without concomitant impaired pain sensation. However, only one such case has ever been reported, and there are no reports on subjective symptoms and detailed somatosensory testing.

**Case presentation:**

Herein, we report the case of a 53-year-old female patient presenting with impaired temperature sensation on the left half of her body, from the neck down, following a small infarction of the right midlateral medulla. The chronological changes in the patient's introspection regarding impairment of thermoception and the results of detailed somatosensory tests, including thermal sense, are shown in this report.

**Conclusions:**

Thorough somatosensory tests, personal descriptions of symptoms, and electrophysiological quantification of similar cases are needed to improve our understanding of the neurological separation of the sensations of pain and temperature at the medullary level.

## Background

Infarctions limited to the lateral medulla can cause various combinations of cerebellar ataxia, vertigo, nystagmus, dysphagia, Horner syndrome, hoarseness, and impaired pain/thermal sensations. Of these symptoms, impaired pain and thermal sensations are the most common. The manifestations include ipsilateral trigeminal–contralateral limb/body, contralateral trigeminal–contralateral limb/body, or bilateral trigeminal–contralateral limb/body involvement; limb/body involvement without trigeminal involvement; or trigeminal involvement without limb/body involvement [1]. Impairment of pain and thermal sensations in the limbs and trunk can be caused by injury to the lateral spinothalamic tract, which runs through the midlateral medulla. On rare occasions, infarctions localised to this region can produce contralateral impairment of these two types of sensations independent of other symptoms [2, 3]. Results of cordotomy [4] and cases of central cord syndrome [5] indicate that at the spinal cord level, the two pathways are doubly dissociated and can be independently impaired. As a result, dissociation of these pathways in the medulla itself has been suggested [6]. To the best of our knowledge, there has only been one report of such a situation: a single case experienced by Arai et al. [7] where a patient presented with isolated impairment of thermoception following a small infarct of the midlateral medulla. However, we cannot know much about the symptoms in this case since the report does not provide details regarding the impairment of thermoception and the results of other somatosensory tests.

Herein, we report the case of a patient presenting with isolated impairment of thermoception following a small infarction of the midlateral medulla. Moreover, we present the patient’s introspective reports of the symptom, as well as detailed quantitative findings from somatosensory testing.

## Case presentation

**Fig. 1:**
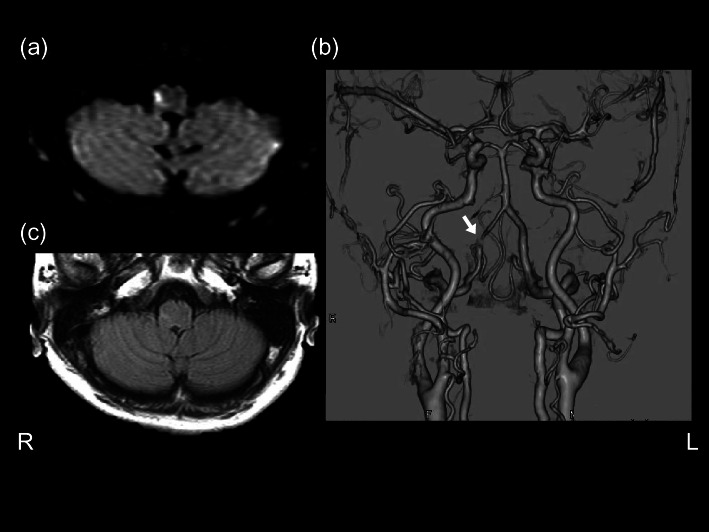
**a** Diffusion-weighted magnetic resonance images at the time of admission revealing a small high-intensity area in the midlateral portion of the right middle medulla. **b** Three-dimensional computed-tomography angiography (3D-CTA) performed 10 days after admission showing stenosis in the right vertebral artery. **c** Fluid-attenuated inversion recovery (FLAIR) MRI conducted 14 days after admission showing a faint high-intensity area almost exactly coinciding with the high-intensity area observed in diffusion-weighted images at admission.

A 53-year-old right-handed woman with a history of smoking and hypertension complained of recurrent ‘coming-and-going’ headaches. After four days of consecutive headaches, she suddenly developed nausea and stagger. Moreover, upon washing her hands, she was completely unable to feel the coldness of the water on her left hand, and when getting into the bath, neither could she feel the water’s warmth on the left side of her body, from the neck down. Following these symptoms, she visited the hospital. Diffusion-weighted magnetic resonance imaging (MRI) of the head revealed a small high-intensity area in the midlateral portion of the right middle medulla (Fig. 1a); no abnormal findings were observed at any other location. The patient was thus admitted with a diagnosis of cerebral infarction, and therapy with aspirin was initiated at 100 mg per os once a day by the seventh day of hospitalisation, ozagrel sodium 40 mg intravenous twice a day by the fifth day of hospitalisation, and clopidogrel 75 mg per os once a day (continued after discharge). Her headache, nausea, and stagger disappeared by the fourth day of hospitalisation, but the thermoanaesthesia persisted, and the patient complained as follows: “When I touch the (steel) bed frame with my left hand, it does not feel cool,” and “When I put water in my hands to wash my face, only my left hand is unable to feel the coldness of the water. Both sides of my face are still able to feel it, so I think my face is okay.” According to the patient, she had never recognised any pain-related sensory abnormalities before or after hospitalisation.

A neurological examination revealed impairment of thermoception in the left upper and lower limbs and trunk. No allodynia was present. However, the patient also reported paraesthesia, stating that “Cold things touching my left leg or trunk sometimes feel hot.” No other abnormalities, such as cerebellar ataxia, vertigo, nystagmus, dysphagia, Horner syndrome, hoarseness, impairment of pain sensation, or impaired facial somatosensory function, were present. Neuropsychological testing revealed no abnormalities related to linguistic function, general attention, general cognition, episodic memory, frontal-lobe function, or construction, and hemispatial neglect was absent (Table 1).

**Table 1:** Results of neuropsychological tests

Test	Performance
**Handedness**	
Edinburgh Handedness Inventory (max: 100)	100
**Language**	
Token test (max: 165)	165
**General attention (short-term memory)**	
Digit span	
Forward	8
Backward	7
Spatial span	
Forward	8
Backward	6
**General cognition**	
Mini-Mental State Examination (max: 30)	29
Raven’s Colored Progressive Matrices (max: 36)	36
**Episodic memory**	
Recall of three words (max: 3)	
Immediate	3
Post-interference	3
She could give accurate oral descriptions of the contents of his previous day's training	
**Frontal function**	
Frontal Assessment Battery (max: 18)	18
Trail Making Test (Japanese version) A (sec.)	27.8
Trail Making Test (Japanese version) B (sec.)	39.8
**Construction**	
Kohs Block Design Test (IQ)	107.2
**Hemispatial neglect**	
Catherine Bergego Scale (max: 30)	0

max Maximum

Three-dimensional computed-tomography angiography (3D-CTA) performed 10 days after admission revealed stenosis in the right vertebral artery (Fig. 1b). Computed tomography (CT) of the cervical spine revealed no abnormalities. A fluid-attenuated inversion recovery (FLAIR) MRI performed 14 days after admission showed a faint high-intensity area almost coinciding with the high-intensity area observed in diffusion-weighted images acquired at admission (Fig. 1c). By this point, the patient’s impaired thermoception had subjectively subsided, and she instead reported a relative difference in the degree of thermoception on the left and right sides of her body, stating “When bathing, the water feels warm on the right side of my body, but not as much on the left,” and “When I touch my bed frame, even though I can somewhat feel its coldness with my left arm, the sensation is weaker than that on my right side.” We performed detailed testing of the patient’s basic and cortical somatosensory modalities [8] (Table 2) and measured somatosensory evoked potentials in both upper limbs.

**Table 2:** Somatosensory Test Results

Tests	Result			
	Left		Right	
	Upper extremity	Lower extremity	Upper extremity	Lower extremity
**a. Basic somatosensory modalities**				
**Pain sensation**	Back of the hand	Back of the foot	Back of the hand	Back of the foot
Threshold (load on pin [g]; range: 1–20)	6	6	6	6
Right-side pain stimulus perceived as equivalent to the left [g] (left side at 8 g)			8	8
**Temperature sensation**	Back of the hand	Back of the foot	Back of the hand	Back of the foot
Discrimination of warm, cold, noxious hot, and noxious cold stimuli (40 °C, 10 °C, 50 °C, 0 °C respectively) Number of positive responses out of three attempts; max: 12)	12	12	12	12
Noxious hot sensation (50 °C)	+	+	+	+
Noxious cold sensation (0 °C)	+	+	+	+
Threshold [°C] (temperature changed at ~0.1 °C/sec. Average of three attempts)				
Threshold for warmth	**38.6**	**36.3**	33.0	32.0
Threshold for cold	28.6	28.3	29.6	27.3
Right-side temperature stimulus perceived equal to the left-side stimulus [°C]				
Left at 25 °C			26	27
Left at 40 °C			**33**	**34**
Left at 10 °C			**18**	**33**
**Tactile sensation**				
Semmes–Weinstein Monofilament [9] (range: 1.65–6.65) ^d^	3.22 at all sites	3.22 at all sites	3.22 at all sites	3.22 at all sites
**Vibratory sense** ^e^	normal	normal	normal	normal
**Joint position sense** (number of correct answers; max: 20) ^f^	20	20	20	20
**b. Cortical somatosensory modality**				
**Two-point discrimination (mm)**				
Stimulation of the middle finger tip	3		3	
Stimulation of the big toe ball		30		20
**Size comparison** (number of correct answers; max: 6) ^g^	6		6	
**Weight comparison** (number of correct answers; max: 6) ^a^	6		6	
**Identification of geometric shapes** (number of correct answers; max: 6) ^b^	6		6	
**Identification of everyday items** (number of correct answers, max: 12) ^c^	12		12	

Abnormal values have been indicated in bold. max: maximum.

^a^ From a group of weights of 10 g, 30 g, 50 g, 70 g, 90 g, 110 g, and 130 g of the same size, the patient received two of adjacent weights (e.g., 10 g and 30 g) to hold in succession and was asked to report which was heavier.

^b^ From a group of shapes consisting of a sphere, circular cone, cylinder, cube, triangular prism, and hexagonal column, the patient received one shape to handle in a location shielded from the patient’s view. Subsequently, all shapes were shown to the patient, and she was asked which shape she had handled.

^c^ The patient was given a ball-point pen, scissors, spoon, clothes peg, golf ball, or stapler to handle in a location shielded from the patient’s view. Then, the patient was asked to name the item.

^d^The ball of the thumb, each fingertip, and the ball of the toe were stimulated.

^e^The patient was asked to report whether a 128 Hz tuning fork was vibrating when touched by it. By gradually reducing the amplitude of the tuning fork, the patient was evaluated for left-right differences of the smallest detectable amplitude. The ulnar styloid and index proximal interphalangeal joint were stimulated.

^f^Joints were moved through approximately 10% of the joint range of motion.

^g^From a group of square leather pieces with side lengths of 40 mm, 45 mm, 50 mm, 55 mm, 60 mm, and 65 mm, the patient received two pieces of adjacent sizes (e.g., 40 mm and 45 mm) to touch in succession and was asked to report which was larger.

**Fig. 2:**
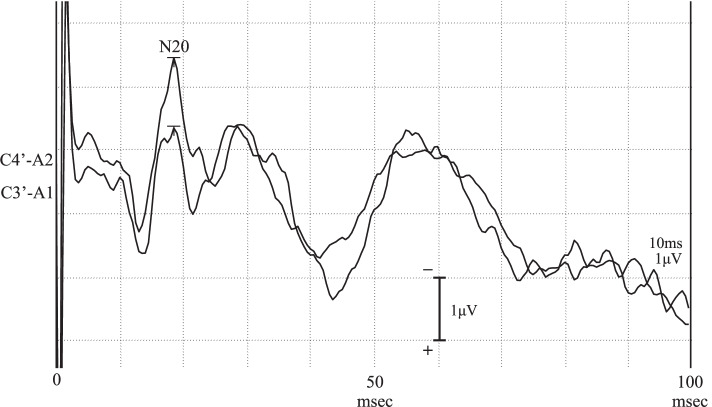
Medial nerve somatosensory evoked potential tests indicating that the N20 distal latency and the P15-N20 amplitude were 18.7 msec/2.086 μV at C3’-A1 and 18.7 msec/2.586 μV at C4’-A2, respectively.

The somatosensory tests were performed with shielding to blind the patient from the stimuli and stimulated body parts, with the patient’s hands manipulating objects. For basic somatosensory modalities, we tested pain sensation, temperature sensation, tactile sense, vibration sense, and joint position sense. Pain and thermoceptive tests were performed on the back of the hands and feet. To investigate pain thresholds, the force being applied to a pin was changed in 1 g increments to determine the minimum load that the patient reported as painful. The results were normal at 6 g for both the left and right upper and lower limbs. Then, while stimulating the left side with a pin with an 8 g load, we changed the load on the right side and asked the patient to report when she felt the same degree of pain as on the left side. The results at the back of the hands and feet were both equal at 8 g. To test thermoception, stimuli at different temperatures (40°C, 10°C, 50°C, and 0°C) were presented to the patient, and she was asked whether these stimuli felt hot, cold, noxiously hot, or noxiously cold, respectively. The patient responded affirmatively on both the right and left sides. However, upon investigating the threshold at which the patient felt a particular stimulus was warm by starting at 30°C and slowly increasing temperature, the results obtained at the back of the left hand and left foot were 5.6°C and 4.3°C higher than the corresponding right-side results, respectively, indicating a clear increase in the threshold. However, when using the same method but instead lowering the temperature to test the threshold for coldness, no clear left-right difference was observed at either the back of the hand or the back of the foot. Different temperatures were presented to the patient’s left side: 25°C as a mildly cold stimulus, 40°C as a warm stimulus, and 10°C as an obviously cold stimulus. Simultaneously, a stimulus starting at 30°C was presented to her right side, and based on her guidance, we sought to find the equivalent temperature on her right side based on her perceived temperature on the left side. We found that at a mildly cold temperature of 25°C, equal responses were observed at both the back of the hand and the back of the foot. However, when presented with a 40°C stimulus on the left side, the patient reported that the right-side stimulus felt identical at a temperature 7°C below that of the left when tested on the back of the right hand and 6°C below that of the left when tested on the back of the right foot. Further, when presented with a 10°C left-side stimulus, the patient reported that the right-side stimulus was identical at a temperature 8°C above that of the left when tested on the back of the right hand and 23°C above that of the left when tested on the back of the right foot. These results indicated that on her left side, the patient was perceiving warm things as considerably colder and cold things as considerably warmer than their actual temperatures. No left-right differences were observed for touch, vibration sense, or joint position sense. For cortical somatosensory modalities, we performed two-point discrimination tests at the middle fingertip and ball of the big toe on the left and right sides, as well as size and weight comparison tests and identification tests of geometric shapes and everyday items with the patient’s left and right hands. Our results indicated that all patient’s cortical somatosensory modalities were normal on both the left and right sides. The test methods for each sensation, other than pain and thermoception, are described in the footnotes of Table 2. In the somatosensory evoked potentials (Fig. 2) by medial nerve stimulation performed 13 days after admission, the N20 distal latency and P15-N20 amplitude were 18.7 ms/2.086 μV at C3’-A1 and 18.7 ms/2.586 μV at C4’-A2, respectively; none of these responses were abnormal.

She was discharged home on the sixteenth day after admission. Her symptoms continued to improve, and at the outpatient visit 10 days after discharge, her only subjective symptom was dysesthesia, which was the feeling of warmth on her left trunk.

## Discussion and conclusions

Based on the early symptom of headache and the findings of head MRI and 3D-CTA, a lateral medulla infarction associated with vertebral artery dissection was diagnosed in this case. In this case, the only neurological symptom was impaired thermoception except for the short-term headache and nausea and the stagger after onset. The lesion was confined to a small area in the midlateral portion of the right middle medulla. Accordingly, our findings are in good agreement with those of Arai et al. [7]. The result of somatosensory evoked potentials indicated that her lesion had not encompassed the medial lemniscus system. Therefore, this case supports Arai’s postulate [7] that only thermoception is impaired when a particular part of the lateral spinothalamic tract running through the midlateral medulla of the medulla oblongata is damaged.

From the patient’s introspective reports, we deduced how her subjective symptoms changed. At the onset, her subjective symptoms consisted of the inability to perceive either high or low temperatures. Two weeks later, her condition had improved to the point that her left side was only relatively worse at perceiving temperatures than her right, and after a month, all symptoms except dysesthesia had disappeared. This case demonstrates that in the case of small infarcts at this site, thermoceptual deficits may improve quickly. The precise reasons for improvement remain unknown. One reason could be that the lesion did not affect the entire thermoceptual pathway in the lateral spinothalamic tract. Another could be that the tissue damage was mild, as reflected in the faintness of the signal in the MRI FLAIR image conducted 14 days after admission.

We could only perform a detailed examination of somatosensory function by the time the patient’s impaired thermoception had improved to a relatively moderate level. However, at that time, we could confirm quantitatively or semi-quantitatively for almost all items that no abnormalities existed other than that in thermoception. In addition, we established that the patient’s sensory disturbance was not an absolute loss of sensations such as hot, cold, noxious hot, and noxious cold, but rather a decrease in sensitivity on the left side relative to the right. We could effectively and quantitatively confirm it matched the patient's introspection. All results obtained from the neuropsychological examination were normal. Therefore, it is unlikely that neuropsychological disorders influenced the patient’s introspective remarks or response to the somatosensory tests.

In this case, hot, cold, noxious hot, and noxious cold were correctly discriminated once the patient’s subjective symptoms had become a matter of relative strength/weakness of perception. Therefore, depending on the time after onset and the degree of disability, a routine neurological examination may not accurately identify damage to the thermoceptual pathway in the lateral spinothalamic tract. Detailed interviews and quantitative sensory tests considering the possibility of thermoceptual disorders are important to avoid overlooking this disorder in patients and correctly evaluate its degree.

This study is subject to some limitations. First, quantitative somatosensory tests were performed only after the patient’s subjective symptoms were largely alleviated. Without collecting data from he onset, we cannot realistically address which kind of introspection corresponds to what kind of test results. Second, our study cannot be used for future comparison since we did not use the standardised quantitative sensory testing developed by the German research network [10]. In the future, we hope that such a standard test will be performed sequentially from the time of onset for similar cases. In addition, our pain and thermoception examinations are based only on the patient’s subjective sensations and lack supporting electrophysiological data. Our observations would have provided further evidence if we performed blink reflex to infer on the region affected [11, 12] and the contact heat [5], cold [13], and laser evoked potentials [14] to discriminate the affected sensations.

Pain and thermoception disorders are doubly dissociated in the spinal cord [4, 5]. However, to the best of our knowledge, there are no case reports where only pain sensation was impaired at the medullary level while thermoception remained intact. The availability of such cases, and the necessary tests, would deepen our understanding of the neurological separation of these two sensations at the medullary level.

## Data Availability

The dataset supporting the conclusions of this article is included within the article.

## Abbreviations

3D-CTA: Three-dimensional computed-tomography angiography
CT: Computed tomography
FLAIR: Fluid-attenuated inversion recovery
MRI: Magnetic resonance imaging
